# Application of an all-in-one snare probe in submucosal tunneling endoscopic resection for an esophageal leiomyoma: the first clinical experience

**DOI:** 10.1055/a-2615-6193

**Published:** 2025-07-01

**Authors:** Jiashaer Bahetinuer, Baohui Song, Xucheng Huo, Ping-Hong Zhou, Mei Liu, Ming-Yan Cai

**Affiliations:** 1 92323 Endoscopy Center and Endoscopy Research Institute, Zhongshan Hospital Fudan University, Shanghai, China; 2 66375 Department of Gastroenterology, Tongji Hospital of Tongji Medical College of Huazhong University of Science and Technology, Wuhan, China

Submucosal tunneling endoscopic resection (STER) establishes a tunnel between the submucosa and muscularis propria proximally to the lesion for en bloc resection, preserving mucosal integrity at the lesion site 1 . Studies have confirmed the efficacy and safety of STER for esophageal leiomyomas 2 . Traditional STER requires multiple instrument exchanges, whereas our all-in-one (AIO, Leomedical, Changzhou, China) device integrates electrosurgical dissection, intraoperative supplementary injection, and snaring, reducing the need for intraprocedural instrument changes ( Fig. 1 ). We herein present a case of AIO-assisted STER for resection of an esophageal leiomyoma.

**Fig. 1:**
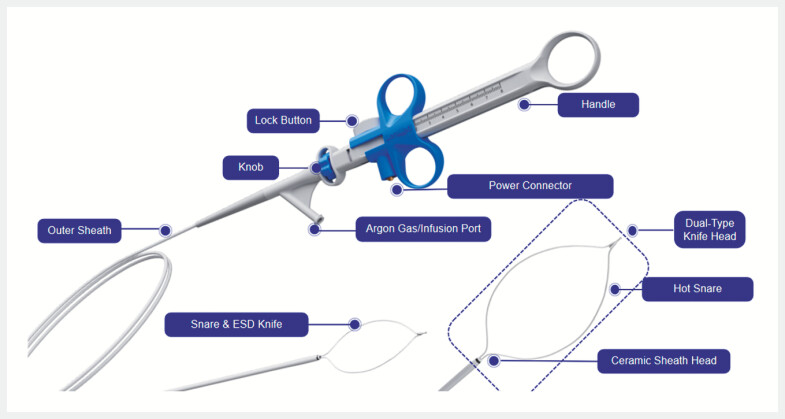
The all-in-one device integrates electrosurgical dissection, intraoperative supplementary injection, and snaring, reducing the need for intraprocedural instrument changes. ESD, endoscopic submucosal dissection.

A 71-year-old woman presenting with retrosternal discomfort for 1 month underwent gastroscopy, which revealed an esophageal submucosal tumor located 21 cm from the incisors ( Fig. 2 ). Further endoscopic ultrasonography revealed a 25 × 20 mm hypoechoic mass originating from the muscularis propria layer. The patient subsequently underwent STER using the AIO device.

**Fig. 2:**
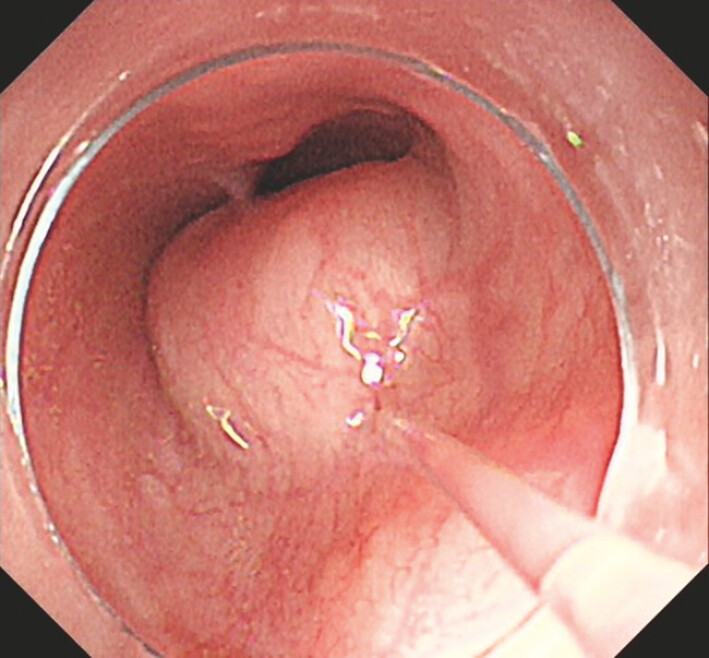
Endoscopic view of the esophageal submucosal tumor at 21 cm from the incisors.

A longitudinal incision was made 2 cm proximally to the lesion using the AIO ESD knife and a submucosal tunnel leading to the lesion was meticulously established until clear visualization of the lesion being continuous with the muscular layer was achieved. To preserve lesion integrity, careful dissection between the tumor and the submucosal layer was performed using the AIO ESD knife, followed by complete resection from the esophageal serosa using an insulation-tipped knife. The intraoperative supplementary injection function of the AIO device facilitated precise tissue plane identification. The resected specimen was successfully retrieved via the AIO snare probe. The mucosal entry was closed with metal clips after hemostasis ( Video 1 ). The total operation time was 37 minutes. The patient experienced no postoperative complications (bleeding, perforation, or fistula) and was discharged after 48 hours of observation. The pathological diagnosis confirmed leiomyoma.

Demonstration of the submucosal tunneling endoscopic resection procedure using the all-in-one device for the resection of an esophageal leiomyoma.Video 1

To the best of our knowledge, this case represents the first successful clinical implementation of the AIO device in STER, demonstrating its convenience, operational efficiency, and safety profile. However, further research is needed.

Endoscopy_UCTN_Code_TTT_1AO_2AG_3AZ

## Notes

Endoscopy E-Videos https://eref.thieme.de/e-videos
*E-Videos* is an open access online section of the journal *Endoscopy* , reporting on interesting cases and new techniques in gastroenterological endoscopy. All papers include a high-quality video and are published with a Creative Commons CC-BY license. Endoscopy E-Videos qualify for HINARI discounts and waivers and eligibility is automatically checked during the submission process. We grant 100% waivers to articles whose corresponding authors are based in Group A countries and 50% waivers to those who are based in Group B countries as classified by Research4Life (see: https://www.research4life.org/access/eligibility/ ). This section has its own submission website at https://mc.manuscriptcentral.com/e-videos .
